# Methanol and Butanol Extracts of* Paeonia lutea* Leaves Repress Metastasis of Squamous Cell Carcinoma

**DOI:** 10.1155/2016/6087213

**Published:** 2016-05-17

**Authors:** Yoshiki Mukudai, Meilin Zhang, Sunao Shiogama, Seiji Kondo, Chihiro Ito, Hiromi Motohashi, Kosuke Kato, Miharu Fujii, Satoru Shintani, Hideyuki Shigemori, Kazunaga Yazawa, Tatsuo Shirota

**Affiliations:** ^1^Department of Oral and Maxillofacial Surgery, School of Dentistry, Showa University, 2-1-1 Kitasenzoku, Ota-ku, Tokyo 145-8515, Japan; ^2^Graduate School of Life and Environmental Sciences, University of Tsukuba, Tsukuba, Ibaraki 305-8572, Japan; ^3^Faculty of Life and Environmental Sciences, University of Tsukuba, Tsukuba, Ibaraki 305-8572, Japan; ^4^Division of Health Food Science, Institute for Nanoscience and Nanotechnology, Waseda University, 2-2 Wakamatsu-cho, Shinjuku-ku, Tokyo 162-0041, Japan

## Abstract

Squamous cell carcinoma (SCC) is one of the most common cancers of the head and neck region worldwide and is generally treated surgically in combination with radiotherapy and/or chemotherapy. However, anticancer agents have numerous serious side effects, and alternative, less toxic agents that are effective as chemotherapeutics for SCC are required. The Paeoniaceae family is widely used in traditional Chinese medicine. We examined methanol and butanol extracts of* Paeonia lutea* (*P. lutea*) leaves for their potential as an anticancer agent. Both extracts decreased the proliferation of SCC cells, induced apoptotic cell death, and modulated migration, adhesion, chemotaxis, and haptotaxis in an extracellular matrix- (ECM-) dependent manner due to altered expression of several integrin subunits. Subsequently, SCC cells were subcutaneously transplanted into athymic nude mice; the extracts reduced the metastasis of SCC cells but had little effect on the volume of the primary tumor or survival or body weight of the mice. The results suggest that the extracts may hold promise for preventing cancer metastasis.

## 1. Introduction

Squamous cell carcinoma (SCC) is one of the most common cancers of the head and neck region worldwide, with more than sixty thousand patients diagnosed each year [1]. Commonly, SCC is treated surgically in combination with radiotherapy and/or chemotherapy. However, treatment outcome is unsatisfactory, with 20% to 50% of patients developing regional recurrence and/or distant metastasis [2]. In addition, anticancer agents often cause a variety of serious side effects; for example,* cis*-platinum (II) diammine dichloride (CDDP), which is often used for chemotherapy of SCC, causes nephrotoxicity [3], neurotoxicity [4], nausea, vomiting [5], ototoxicity [6], and xerostomia (dry mouth) [7, 8]. Hence, less toxic chemotherapeutics for SCC are required.

The Paeoniaceae family is widely used in traditional Chinese medicine. Previous studies reported that the root barks of Paeoniaceae are used as therapeutics for various diseases such as diabetes [9], Alzheimer's disease [10, 11], arthritis [12], inflammation [13], sepsis [14], brain-ischemia-reperfusion injury [15], and virus infections [16, 17]. Moreover, several studies have reported anticancer effects of Paeoniaceae family preparations [18–23].

Traditional Chinese medicine has been used to treat a variety of diseases for several thousands of years; therefore, we launched an interinstitutional collaborative project in 2010 to evaluate the therapeutic potential of herbal extracts for disorders of the head and neck region [23–26]. Here, we examined methanol and butanol extracts of* Paeonia lutea* (*P. lutea*) leaves for their potential as an anticancer agent. Our results indicate that these extracts modulate the migration and adhesion of SCC cells to the extracellular matrix (ECM) by altering integrin subunit expression* in vitro* and reduce metastasis of the cells* in vivo*; however, transplanted tumor growth and survival of the recipient animals were essentially unaffected.

## 2. Materials and Methods

### 2.1. Plant Materials and Preparation of Plant Extracts

*P. lutea* leaves were collected from Tsukuba Peony Garden (Tsukuba, Ibaraki, Japan) in October 2012. A voucher specimen (UTHS1210) was deposited at the Laboratory of Natural Products Chemistry, Graduate School of Life and Environmental Sciences, University of Tsukuba. The leaves were air dried for 2 days at room temperature (dry weight, 350 g) and then extracted with methanol (4 L) for 1 week. The extraction was repeated once. The two methanol extracts were combined and concentrated* in vacuo* at 38°C to give the methanol extract (ME, 80.1 g). The extract was partitioned with ethyl acetate three times (800 mL each) and H_2_O (800 mL). The ethyl acetate was evaporated to afford ethyl acetate soluble materials (EA, 28.5 g). The H_2_O layer was partitioned with butanol three times (600 mL each) to give butanol-soluble (BU, 20.5 g), butanol-insoluble (BW, 2.1 g), and H_2_O-soluble materials (W, 16.5 g). Finally, aliquots of each of these materials were dissolved in phosphate-buffered saline (PBS) at a concentration of 1 mg/mL and sterilized by passage through a Millex syringe filter (Merck Millipore, Billerica, MA).

### 2.2. Cell Culture and Anchorage-Independent Growth Assay

SAS cells, a human oral SCC cell line [27], were cultured in high-glucose Dulbecco's Modified Eagle Medium (DMEM; Wako, Osaka, Japan) supplemented with 10% fetal bovine serum (FBS) and penicillin-streptomycin solution (Sigma-Aldrich, St. Louis, MO) at 37°C, 5% CO_2_, and 100% humidity. Anchorage-independent growth assay was carried out using a commercial kit (Cytoselect 96-Well* In Vitro* Tumor Sensitivity Assay kit, Cell Biolabs, San Diego, CA) as described previously [28].

### 2.3. Cell Growth and Apoptosis Assays

One thousand cells were seeded into each well of a 96-well tissue culture plate. After 48 h, the cells were assayed using the tetrazolium salt 3-[4,5-dimethylthiazol-2-yl]-2,5-diphenyltetrazolium bromide (MTT) assay, as described previously [29]. The activities of caspases 3/7, 8, and 9 were measured using a Caspase-Glo (Promega, Madison, WI) and GloMax-Multi+ Detection System (Promega) according to the manufacturer's protocol. Genomic DNA fragmentation was investigated using a commercial kit (Apopladder EX; Takara, Shiga, Japan) according to the manufacturer's protocol.

### 2.4. Protein Preparation and Western Blotting Analysis

Total cellular protein was prepared as described previously [30]. Protein concentration was measured using Quick Start Bradford Reagent (Bio-Rad, Hercules, CA) using bovine serum albumin (BSA) as a standard. Protein aliquots were stored at −80°C until use. For Western blot analysis, 20 *μ*g of total cellular protein was subjected to sodium dodecyl sulfate polyacrylamide electrophoresis (SDS-PAGE) on a 4% to 20% gradient gel (Bio-Rad); then the blot was transferred onto a polyvinylidene difluoride membrane (Life Technologies, Carlsbad, CA). Blocking, 1st and horseradish-peroxidase-conjugated 2nd antibody reactions and washing were conducted as previously described [28]. The chemiluminescence signals were visualized using Amersham ECL Western Blotting Detection Reagents (GE Healthcare UK Ltd., Buckinghamshire, UK) and ChimiDoc XRS Plus ImageLab System (Bio-Rad). The 1st and 2nd antibodies were purchased from common suppliers.

### 2.5. Adhesion, Wound Healing, and Migration Assays

The cells were grown in a monolayer culture in the presence or absence of the various* P. lutea* leaf extracts. After 3 d, the cells were assayed. Adhesion and wound healing assays were carried out using commercial kits (CytoSelect 48-Well Cell Adhesion Assay, CytoSelect 24-Well Wound Healing Assay, Cell Biolabs) according to the manufacturer's protocol. To assay migration, a chemotaxis chamber containing a membrane with 8 mm pores (Chemotaxicell, Kurabo, Osaka Japan) was coated with 50 *μ*g/mL bovine type I collagen (Koken, Tokyo, Japan) or 50 *μ*g/mL bovine fibronectin (Sigma-Aldrich) according to the manufacturer's protocol and was set into each well of a 24-well culture plate. After drying the Chemotaxicell membrane, 500 *μ*L of DMEM with or without 10% FBS was added to the bottom of the well as a chemoattractant, 200 *μ*L of DMEM containing 2 × 10^4^ cells was added to the chamber, then the plate was incubated for 24 h at 37°C, 5% CO_2_, and 100% humidity. The membranes were then washed, removed from the chamber, fixed with 4% formaldehyde (Wako, Osaka, Japan), and stained with crystal violet (Wako). The membranes were examined under an optical microscope and photographs (×400) were taken.

### 2.6. Animals

The care and treatment of the experimental animals complied with the Showa University Guidelines for Animal Experiments, and the experimental protocol was approved by the Animal Experimentation Committee of Showa University. The dorsal flank of four-week-old female athymic Balb/c* nu/nu* mice (CLEA Japan, Tokyo) was subcutaneously injected with a PBS-suspension of 1 × 10^6^ SAS cells, as described previously [30]. After 1 week, tumor formation was measured (approximately 10 mm^3^), and the mice were divided into 6 groups (control, ME, EA, BU, BW, and W). Therefore, the 7th day after SAS cell injection was designated “day 1,” thereafter. Each herbal extract was suspended in PBS at the concentration of 2 mg/mL and 100 mg/kg body weight was orally administrated using a sterilized feeding needle once every three days for 40 or 70 days. In the control group, only PBS (0.1 mL) was administrated. The body weight and diameters (large and small) of each mouse were measured and the tumor volume was determined by direct measurement and calculated using the formula [30] *π*/6 × (large diameter) × (small diameter)^2^. On day 40 or 70, the mice were sacrificed, and the tumors together with the surrounding soft tissue, liver, and lungs were harvested for histochemical analysis.

### 2.7. Histochemistry and Immunohistochemistry

The tissue was fixed, embedded, sliced, and subjected to hematoxylin-eosin (HE) staining as described previously [31]. Immunohistochemistry was conducted as described previously [31]. After 24 h incubation with a 1/50 dilution of primary antibody for human cytokeratin 10/13 (Santa Cruz Biotechnology, Santa Cruz, CA), the slide was incubated with Simple Stain MAX-PO (Nichirei, Tokyo, Japan) and visualized using Envision HRP/Kit (Dako, Kyoto, Japan); the manufacturer's suggested protocol for each commercial kit was used.

### 2.8. Statistical Analysis

Unless otherwise specified, all experiments were repeated at least* twice*, and similar results were obtained in the repeat experiments. Statistical analysis for mouse survival was determined by the log rank test. Other statistical analyses were carried out using two-tailed, unpaired Student's *t*-test. Data are expressed as means ± standard deviation of at least three data items. A *p* value < 0.05 was considered significant.

## 3. Results

### 3.1. Extracts of* P. lutea* Leaves Decreased Proliferation of SCC Cells in an Anchorage-Independent Manner, but No Effect Was Observed on Monolayer Cultures

**Figure 1:**
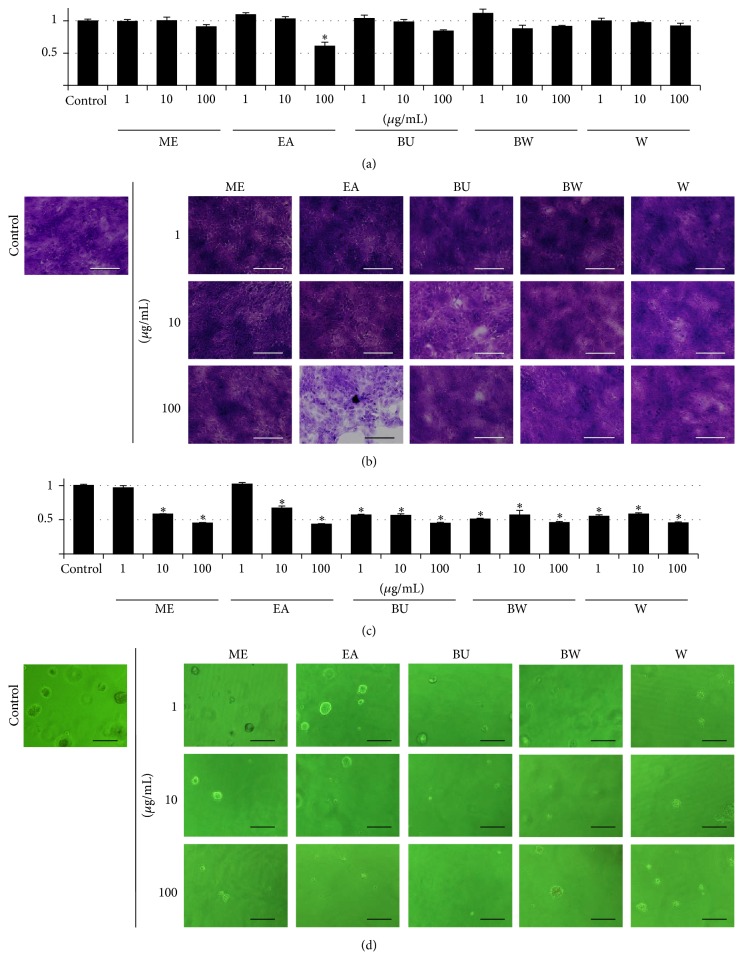
Growth-inhibitory effects of extracts of* P. lutea* leaves. SAS cells were grown in a monolayer ((a) and (b)) or on soft agar ((c) and (d)) culture. Methanol (ME), ethyl acetate (EA), butanol (BU), butanol-insoluble (BW), and water (W) extracts of* P. lutea* leaves were added to the culture medium at 0 (control) and 1, 10, or 100 *μ*g/mL. After 7 d, the cells were subjected to MTT assay ((a) and (c)) and crystal violet staining (b) or examined under a phase-contrast microscope (d). Data in (a) and (c) are means ± standard deviations of 3 cultures; the mean of the control cultures is taken as “1.” ^*∗*^ *p* < 0.05 versus control. Bars, 100 *μ*m (b) and 200 *μ*m (d).

We first investigated the effects of the* P. lutea* leaf extracts on the proliferation of SCC cells (Figure 1). Only 100 *μ*g/mL EA had a significant effect on monolayer cultures ((a) and (b)). In contrast, all extracts showed a significant growth-inhibitory effect on soft agar cultures using the MTT assay (c). Importantly, phase-microscopy images (d) indicated that all the extracts decreased both the number and size of SCC cells compared to the control culture. These results suggest that extracts of* P. lutea* leaves are likely to reduce both the proliferation and malignancy of SCC cells.

### 3.2. Extracts of* P. lutea* Leaves Induce Apoptotic Cell Death of SCC Cells in Soft Agar Culture

**Figure 2:**
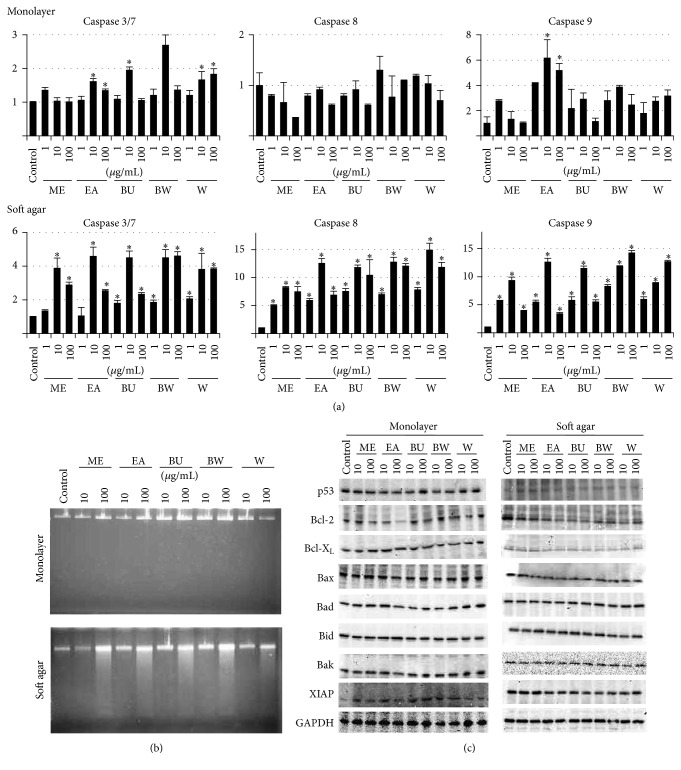
Apoptosis-induced effects of* P. lutea* leaf extracts. SAS cells were grown in a monolayer or on soft agar culture. Methanol (ME), ethyl acetate (EA), butanol (BU), butanol-insoluble (BW), and water (W) extracts of* P. lutea* leaves were added to the culture medium at 1 to 100 *μ*g/mL ((a) and (b)), 10 to 100 *μ*g/mL (c), or 0 *μ*g/mL (control). After 3 d, the cells were subjected to caspase 3/7, 8, and 9 assays (a), a DNA ladder assay (b), or Western blotting analysis for apoptosis-related proteins (p53, Bcl-2, Bcl-X_L_, Bax, Bad, Bid, Bak, and XIAP) using GAPDH as an internal control (c). Data in (a) are means ± standard deviations of 3 cultures; the mean of the control cultures is taken as “1.” ^*∗*^ *p* < 0.05 versus control.

We tested the hypothesis that the reduced MTT activity was due to apoptotic cell death by investigating the apoptotic effects of the extracts (Figure 2). Caspase activity assays (a) revealed that 10–100 mg/mL of EA significantly increased caspase 3/7 and 9 activities in a monolayer culture, whereas all the extracts strongly increased caspase activities in a soft agar culture. On the other hand, a DNA ladder assay (b) showed almost no DNA fragmentation in monolayer cultures treated with any of the extracts, whereas significant DNA fragmentation was observed in all the test soft agar cultures, in disagreement with the caspase assays. Furthermore, we examined the effects of the extracts on modulating apoptosis-related protein expressions by Western blotting analysis (c). In both monolayer and soft agar cultures, EA and BU reduced the expression of Bcl-2, a mitochondrial antiapoptotic protein, but the expression of other proteins (p53, Bcl-X_L_, Bax, Bad, Bid, Bak, and XIAP) was essentially unaltered. These results indicate that EA and BU might affect the mitochondrial apoptotic pathway, in an anchorage-independent manner.

### 3.3. Extracts of* P. lutea* Leaves Decreased the Adhesion of ECM

**Figure 3:**
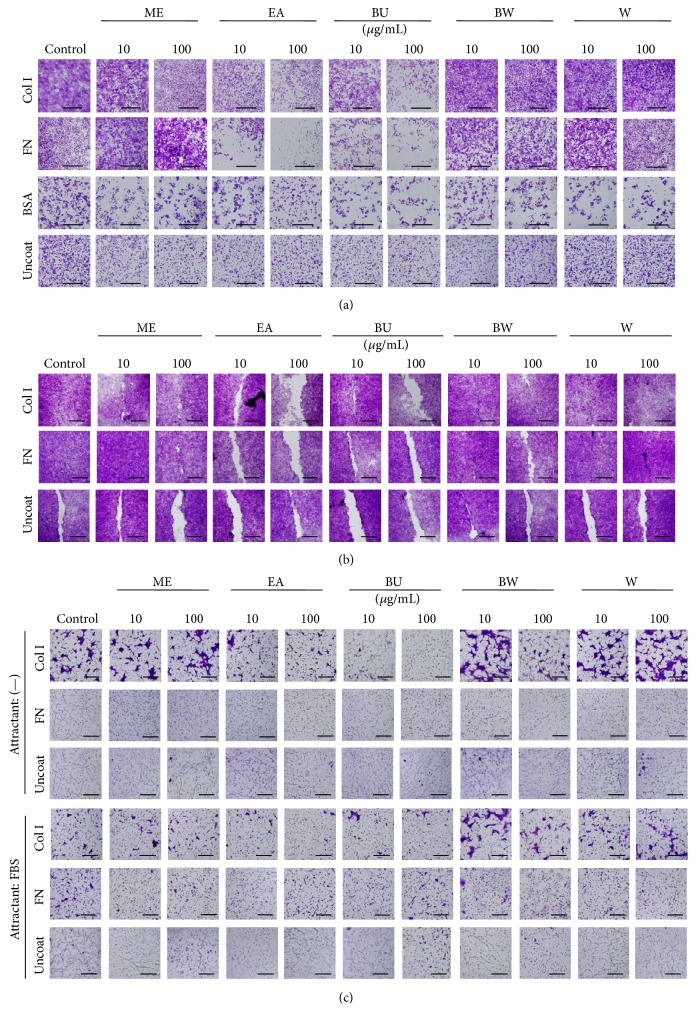
Effects of* P. lutea* leaf extracts on cell adhesion to ECMs. SAS cells were grown in a monolayer culture in the presence or absence (control) of methanol (ME), ethyl acetate (EA), butanol (BU), butanol-insoluble (BW), and water (W) extracts of* P. lutea* leaves at 10 to 100 *μ*g/mL. After 3 d, the cells were subjected to adhesion (a), wound healing (b), and migration (c) assays for ECM (type I collagen (Col I), fibronectin (FN), and BSA) or an uncoated surface (Uncoat). 10% FBS was used as a chemoattractant or not (—) in the migration assay (c). Bars, 100 *μ*m (a and c) and 1 mm (b).

Next, we examined the effects of adhesion, migration, and invasion in an ECM-dependent manner (Figure 3). Cell adhesion assays (a) showed that EA and BU reduced the attachment of SCC cells to type I collagen (Col I) and fibronectin (FN); similar results regarding cell migration were obtained using a cell migration assay (b). Furthermore, a cell invasion assay (c) showed that EA and BA reduced SAS cell invasion of Col I and FN and that the effects were independent of a chemoattractant. Those results imply that EA and BU extracts decrease both chemotaxis and haptotaxis of SCC cells through the ECM, thereby potentially preventing the invasion and metastasis of SCC cells.

### 3.4. Extracts of* P. lutea* Leaves Decreased the Expression of Several Integrin Subunits

**Figure 4:**
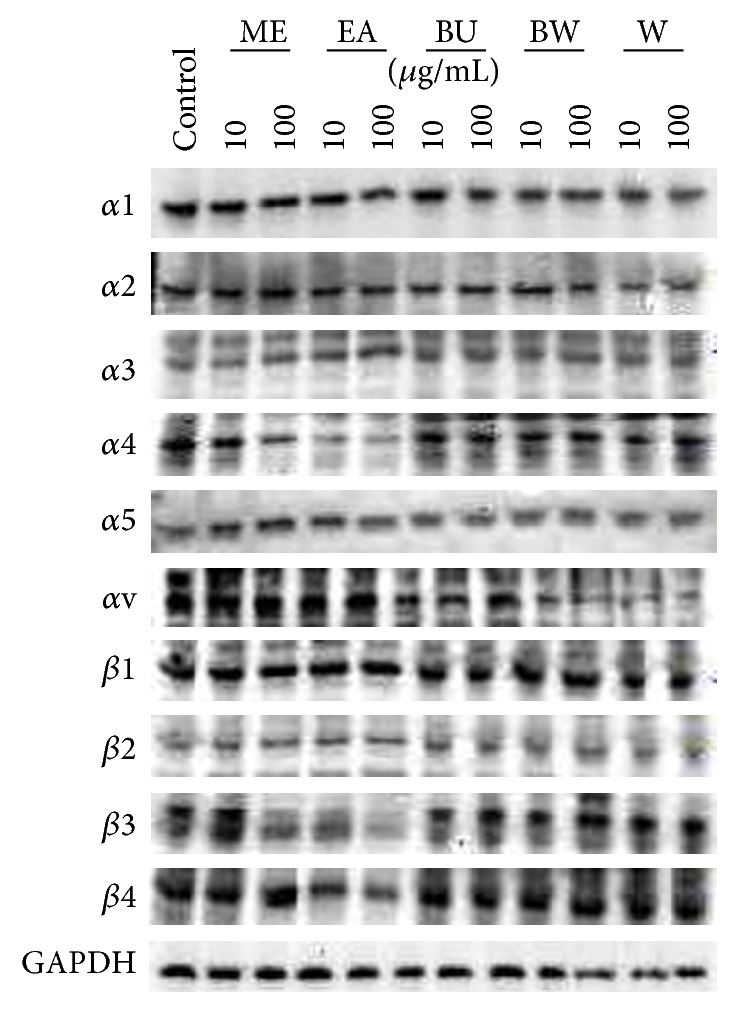
Effects of* P. lutea* leaf extracts on expression of integrin proteins. SAS cells were grown in a monolayer culture in the presence or absence (control) of methanol (ME), ethyl acetate (EA), butanol (BU), butanol-insoluble (BW), and water (W) extracts of* P. lutea* leaves at 10 to 100 *μ*g/mL. After 3 d, total cellular proteins were purified and 20 *μ*g aliquots were subjected to Western blotting for *α*1, *α*2, *α*3, *α*4, *α*5, *α*v, *β*1, *β*2, *β*3, and *β*4 integrins. GAPDH was used as an internal control.

The adhesion, migration, and invasion of various cells, including cancer cells, rely on integrin proteins. We therefore investigated whether the extracts modulate the expression of major integrin subunits. Western blotting analysis indicated that BW and W had little effect on the expression of integrin subunits (Figure 4): the expression of *α*4, *β*3, *β*4, and *β*5 integrin subunits was attenuated by EA and BU, but expression of the other subunits was essentially unchanged. These results suggest that the altered response of SAS cells to the ECM is at least partly due to modulation of the expression of several integrin subunits.

### 3.5. Extracts of* P. lutea* Reduce Metastasis of SCC Cells

**Figure 5:**
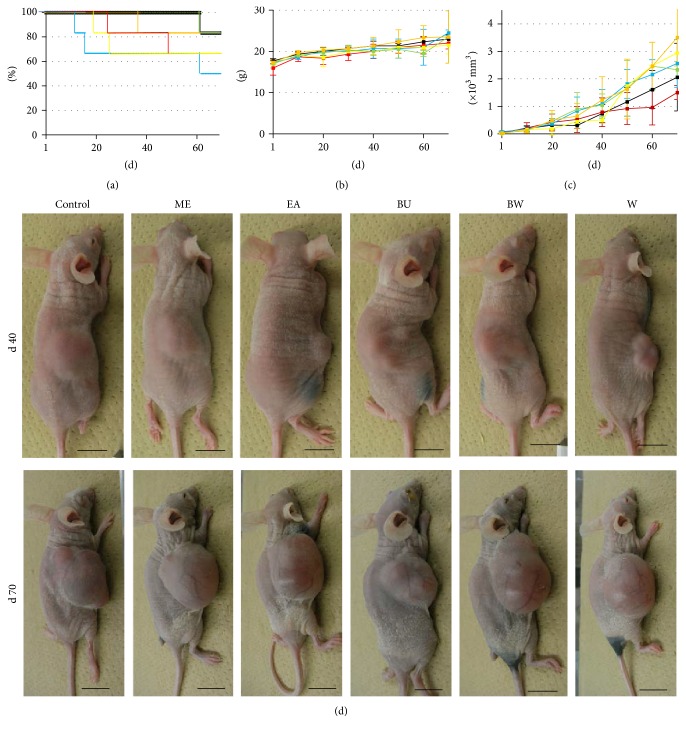
Effects of* P. lutea* leaf extracts on mouse survival and tumor growth in SAS-cell-xenograft nude mice. One million SAS cells were subcutaneously injected into the dorsal flank of nude mice (see Section 2). After 7 d, the mice were divided into 6 groups: vehicle (black lines in (a), (b), and (c) and control in (d)) methanol (red lines and ME), ethyl acetate (blue lines and EA), butanol (yellow lines and BU), butanol-insoluble (green lines and BW), and water (orange lines and W) extracts of* P. lutea* leaves were administrated once every three days at a concentration of 100 mg/kg body weight in PBS. Mouse survival Kaplan Meier plot in %, body weight in grams, and tumor volume in ×10^3^ mm^3^ of each group are depicted in (a), (b), and (c), respectively; images of a representative mouse in each group were taken at d 40 and 70 (d). Bars, 1 cm.

**Figure 6:**
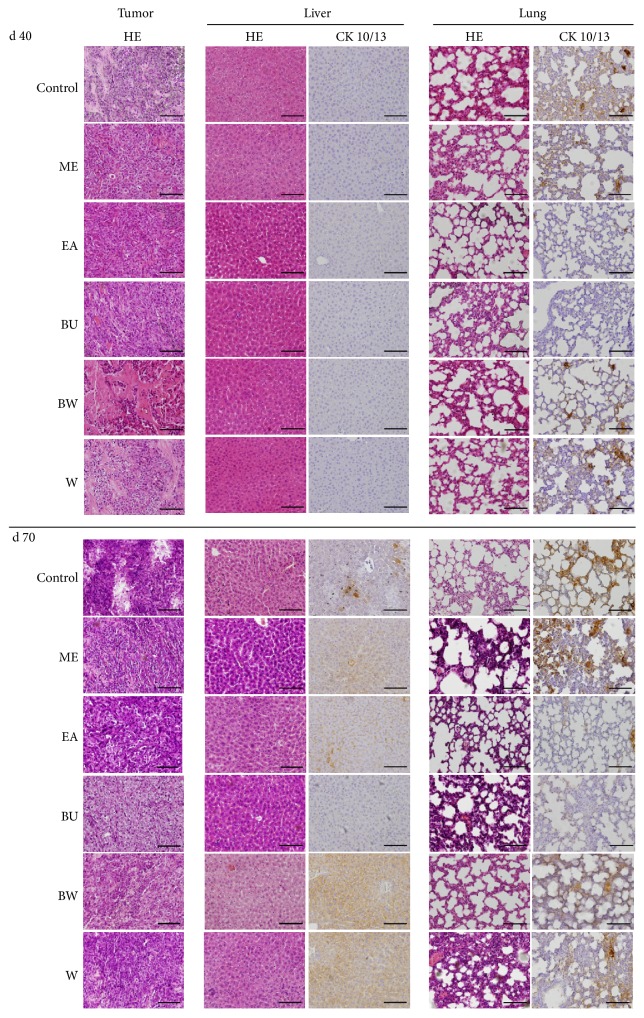
Effects of* P. lutea* leaf extracts on hepatic and pulmonary tumor-cell-metastasis in SAS-cell-xenograft nude mice. One million SAS cells were subcutaneously injected into the dorsal flank of nude mice, and vehicle (control), methanol (ME), ethyl acetate (EA), butanol (BU), butanol-insoluble (BW), and water (W) extracts of* P. lutea* leaves were administrated once every three days at 100 mg/kg body weight in PBS (see Section 2). After d 40 or 70, the mice were sacrificed, and the tumors, livers, and lungs were fixed and subjected to HE staining (HE) and immunohistochemistry for cytokeratin 10/13 (CK 10/13). Bars, 200 *μ*m.

The results of the above* in vitro* experiments prompted us to investigate* in vivo* whether the extracts might be useful as a novel anticancer medicine. Each extract was orally administrated to SCC-transplanted nude mice (Figure 5). Contrary to our expectation, neither survival (a), body weight (b), nor tumor growth ((c) and (d)) was affected by the extracts compared to the control group. Furthermore, histochemical analysis (Figure 6) showed that all extracts had little effect on the primary tumor. Liver and lung tissues were examined to determine the effects of the extracts on metastasis. HE staining showed no obvious metastatic tumor cells. Immunohistochemistry for cytokeratin 10/13, a representative marker of SCC cells, showed positive cells in the control and ME, BW, and W extracts mice, indicating micrometastasis from the primary tumor. Interestingly, however, administration of EA and BU extracts significantly reduced the number of positive cells. These results indicate that EA and BU extracts of* P. lutea* exhibit pharmacological effects to decrease hepatic and pulmonary metastasis from epidermal SCC and suggest that the extracts may hold promise as anticancer therapeutics.

## 4. Discussion

The Paeoniaceae family has been widely used in traditional Chinese medicine for thousands of years. Recent studies have revealed that extracts of* Paeoniaceae* may provide alternative anticancer therapeutics [18–23] and prompted us to investigate the extracts of* P. lutea* leaves for their anticancer potency. We first conducted* in vitro* studies to determine the effects of* P. lutea* leaf extract on proliferation (Figure 1) and apoptosis (Figure 2) of SCC cells. The extracts decreased proliferation and induced apoptotic cell death mainly by a mitochondrial signaling pathway, similar to Paeoniaceae root extracts [32]. Interestingly, the effects were more prominent in anchorage-independent cultures. Since proliferation and migration in an anchorage-independent manner reflect properties of malignant cancer cells [33], those results suggested that the extracts could be useful as an anticancer agent.

We next examined whether the extracts modulate adhesion to ECM and affect ECM-dependent migration, chemotaxis, and haptotaxis (Figure 3). EA and BU extracts significantly decreased adhesion to Col I and fibronectin. Several previous studies have reported that adhesion to ECM plays an important role in the invasion and metastasis of cancer cells [33, 34]. Thus, the present results suggest that the extracts might also decrease invasion and metastasis. Furthermore, adhesion between ECM and normal and cancer cells is supported by integrins, in particular *α*5*β*1 integrin, a main component of the fibronectin receptor [35]. However, Western blotting analysis for various major integrin subunits (Figure 4) showed essentially no modulation of the expression of *α*5 or *β*1 subunits by the extracts, although *α*4, *β*3, *β*4, and *β*5 integrin subunit expression was suppressed by the EA and BU extracts. Further studies, for example, on integrin-dependent signaling pathways and on other adhesion molecules, are required to resolve this discrepancy. Nevertheless, the present study demonstrated that EA and BU extracts modulate the expression of several integrin subunits, thus decreasing the invasion and metastasis of SCC cells* in vitro*.

The potential utility of the extracts for clinical use was investigated by an animal experiment and subsequent histochemical examination. A dose of 100 mg/kg body weight was chosen based on our previous studies [36–38]. The extracts were orally administrated to SAS-cell-transplanted nude mice, and the survival and body weight of the mice and the volume of the primary tumor were measured (Figure 5). In contrast to the* in vivo* results, no antitumor effects were observed. HE staining and subsequent histochemical examination of the primary tumor, liver, and lung (Figure 6) showed no effect of the extracts. Cytokeratin 10/13 is highly expressed in SCC cells [39, 40], including SAS cells, and immunohistochemistry for cytokeratin 10/13 is indicative of metastasis of tumor cells in liver and lung. Mice given the extracts for 70 days showed positive cells in the organ (liver and lung). However, administration of EA and BU extracts significantly decreased the number and size of cytokeratin 10/13-positive tumor cells, suggesting that the extracts may have potency for reducing hepatic and pulmonary metastasis of epidermal SCC.

Our results demonstrate that EA and BU extracts of* P. lutea* have pharmacological effects of preventing the metastasis of SCC cells. The chemical composition, side effects, and minimum required dosage of the extracts of the extraction are currently being investigated in detail and will be reported in the near future.

## References

[1] Machiels J.-P., Lambrecht M., Hanin F.-X. (2014). Advances in the management of squamous cell carcinoma of the head and neck. *F1000Prime Reports*.

[2] Lin Y.-C., Chen H.-W., Kuo Y.-C., Chang Y.-F., Lee Y.-J., Hwang J.-J. (2010). Therapeutic efficacy evaluation of curcumin on human oral squamous cell carcinoma xenograft using multimodalities of molecular imaging. *American Journal of Chinese Medicine*.

[3] dos Santos N. A. G., Rodrigues M. A. C., Martins N. M., dos Santos A. C. (2012). Cisplatin-induced nephrotoxicity and targets of nephroprotection: an update. *Archives of Toxicology*.

[4] McWhinney S. R., Goldberg R. M., McLeod H. L. (2009). Platinum neurotoxicity pharmacogenetics. *Molecular Cancer Therapeutics*.

[5] Herrstedt J. (2008). Antiemetics: an update and the MASCC guidelines applied in clinical practice. *Nature Clinical Practice Oncology*.

[6] Rybak L. P., Mukherjea D., Jajoo S., Ramkumar V. (2009). Cisplatin ototoxicity and protection: clinical and experimental studies. *Tohoku Journal of Experimental Medicine*.

[7] Rapidis A. D., Trichas M., Stavrinidis E. (2006). Induction chemotherapy followed by concurrent chemoradiation in advanced squamous cell carcinoma of the head and neck: final results from a phase II study with docetaxel, cisplatin and 5-fluorouracil with a four-year follow-up. *Oral Oncology*.

[8] Psyrri A., Kwong M., DiStasio S. (2004). Cisplatin, fluorouracil, and leucovorin induction chemotherapy followed by concurrent cisplatin chemoradiotherapy for organ preservation and cure in patients with advanced head and neck cancer: long-term follow-up. *Journal of Clinical Oncology*.

[9] Lau C. H., Chan C. M., Chan Y. W. (2007). Pharmacological investigations of the anti-diabetic effect of Cortex Moutan and its active component paeonol. *Phytomedicine*.

[10] Fujiwara H., Tabuchi M., Yamaguchi T. (2009). A traditional medicinal herb Paeonia suffruticosa and its active constituent 1,2,3,4,6-penta-O-galloyl-*β*-d-glucopyranose have potent anti-aggregation effects on Alzheimer's amyloid *β* proteins in vitro and in vivo. *Journal of Neurochemistry*.

[11] Zhou J., Zhou L., Hou D., Tang J., Sun J., Bondy S. C. (2011). Paeonol increases levels of cortical cytochrome oxidase and vascular actin and improves behavior in a rat model of Alzheimer's disease. *Brain Research*.

[12] Kim H. S., Kim A.-R., Lee J. M. (2012). A mixture of Trachelospermi caulis and Moutan cortex radicis extracts suppresses collagen-induced arthritis in mice by inhibiting NF-*κ*B and AP-1. *Journal of Pharmacy and Pharmacology*.

[13] Chou T.-C. (2003). Anti-inflammatory and analgesic effects of paeonol in carrageenan-evoked thermal hyperalgesia. *British Journal of Pharmacology*.

[14] Li G., Seo C.-S., Lee K.-S. (2004). Protective constituents against sepsis in mice from the root cortex of *Paeonia suffruticosa*. *Archives of Pharmacal Research*.

[15] Hsieh C.-L., Cheng C.-Y., Tsai T.-H. (2006). Paeonol reduced cerebral infarction involving the superoxide anion and microglia activation in ischemia-reperfusion injured rats. *Journal of Ethnopharmacology*.

[16] Hsiang C.-Y., Hsieh C.-L., Wu S.-L., Lu L., Lai T.-Y., Ho (2001). Inhibitory effect of anti-pyretic and anti-inflammatory herbs on herpes simplex virus replication. *American Journal of Chinese Medicine*.

[17] Au T. K., Lam T. L., Ng T. B., Fong W. P., Wan D. C. C. (2001). A comparison of HIV-1 integrase inhibition by aqueous and methanol extracts of Chinese medicinal herbs. *Life Sciences*.

[18] Hung J.-Y., Yang C.-J., Tsai Y.-M., Huang H.-W., Huang M.-S. (2008). Antiproliferative activity of paeoniflorin is through cell cycle arrest and the Fas/Fas ligand-mediated apoptotic pathway in human non-small cell lung cancer A549 cells. *Clinical and Experimental Pharmacology and Physiology*.

[19] Choi H. S., Seo H.-S., Kim J. H., Um J.-Y., Shin Y. C., Ko S.-G. (2012). Ethanol extract of paeonia suffruticosa Andrews (PSE) induced AGS human gastric cancer cell apoptosis via fas-dependent apoptosis and MDM2-p53 pathways. *Journal of Biomedical Science*.

[20] Xing G., Zhang Z., Liu J., Hu H., Sugiura N. (2010). Antitumor effect of extracts from moutan cortex on DLD-1 human colon cancer cells in vitro. *Molecular Medicine Reports*.

[21] Wang S.-C., Tang S.-W., Lam S.-H. (2012). Aqueous extract of Paeonia suffruticosa inhibits migration and metastasis of renal cell carcinoma cells via suppressing VEGFR-3 pathway. *Evidence-Based Complementary and Alternative Medicine*.

[22] Lin M.-Y., Lee Y.-R., Chiang S.-Y. (2013). Cortex Moutan induces bladder cancer cell death via apoptosis and retards tumor growth in mouse bladders. *Evidence-Based Complementary and Alternative Medicine*.

[23] Li C., Yazawa K., Kondo S. (2012). The root bark of Paeonia moutan is a potential anticancer agent in human oral squamous cell carcinoma cells. *Anticancer Research*.

[24] Mukudai Y., Kondo S., Koyama T. (2014). Potential anti-osteoporotic effects of herbal extracts on osteoclasts, osteoblasts and chondrocytes in vitro. *BMC Complementary and Alternative Medicine*.

[25] Sato D., Kondo S., Yazawa K. (2013). The potential anticancer activity of extracts derived from the roots of Scutellaria baicalensis on human oral squamous cell carcinoma cells. *Molecular and Clinical Oncology*.

[26] Mukudai Y., Kondo S., Shiogama S. (2013). Root bark extracts of *Juncus effusus* and *Paeonia suffruticosa* protect salivary gland acinar cells from apoptotic cell death induced by cis-platinum (II) diammine dichloride. *Oncology Reports*.

[27] Okumura K., Konishi A., Tanaka M., Kanazawa M., Kogawa K., Niitsu Y. (1996). Establishment of high- and low-invasion clones derived for a human tongue squamous-cell carcinoma cell line SAS. *Journal of Cancer Research and Clinical Oncology*.

[28] Mukudai Y., Kondo S., Fujita A., Yoshihama Y., Shirota T., Shintani S. (2013). Tumor protein D54 is a negative regulator of extracellular matrix-dependent migration and attachment in oral squamous cell carcinoma-derived cell lines. *Cellular Oncology*.

[29] Tsukamoto H., Kondo S., Mukudai Y. (2011). Evaluation of anticancer activities of benzo[c]phenanthridine alkaloid sanguinarine in oral squamous cell carcinoma cell line. *Anticancer Research*.

[30] Yasuda A., Kondo S., Nagumo T. (2011). Anti-tumor activity of dehydroxymethylepoxyquinomicin against human oral squamous cell carcinoma cell lines in vitro and in vivo. *Oral Oncology*.

[31] Shiogama S., Yoshiba S., Soga D., Motohashi H., Shintani S. (2013). Aberrant expression of EZH2 is associated with pathological findings and P53 alteration. *Anticancer Research*.

[32] Brenner D., Mak T. W. (2009). Mitochondrial cell death effectors. *Current Opinion in Cell Biology*.

[33] Paoli P., Giannoni E., Chiarugi P. (2013). Anoikis molecular pathways and its role in cancer progression. *Biochimica et Biophysica Acta—Molecular Cell Research*.

[34] Indran I. R., Tufo G., Pervaiz S., Brenner C. (2011). Recent advances in apoptosis, mitochondria and drug resistance in cancer cells. *Biochimica et Biophysica Acta—Bioenergetics*.

[35] Rathinam R., Alahari S. K. (2010). Important role of integrins in the cancer biology. *Cancer and Metastasis Reviews*.

[36] Shirosaki M., Koyama T., Yazawa K. (2012). Apple leaf extract as a potential candidate for suppressing postprandial elevation of the blood glucose level. *Journal of Nutritional Science and Vitaminology*.

[37] Shirosaki M., Goto Y., Hirooka S., Masuda H., Koyama T., Yazawa K. (2012). Peach leaf contains multiflorin A as a potent inhibitor of glucose absorption in the small intestine in mice. *Biological and Pharmaceutical Bulletin*.

[38] Koyama T., Nakajima C., Nishimoto S., Takami M., Woo J.-T., Yazawa K. (2012). Suppressive effects of the leaf of *Terminalia catappa* L. on osteoclast differentiation in vitro and bone weight loss in vivo. *Journal of Nutritional Science and Vitaminology*.

[39] Soni S., Mathur M., Shukla N. K., Deo S. V. S., Ralhan R. (2003). Stromelysin-3 expression is an early event in human oral tumorigenesis. *International Journal of Cancer*.

[40] Qiu C., Wu H., He H., Qiu W. (2003). A cervical lymph node metastatic model of human tongue carcinoma: serial and orthotopic transplantation of histologically intact patient specimens in nude mice. *Journal of Oral and Maxillofacial Surgery*.

